# Method of Predicting the Crimp of Jacquard-Woven Fabrics

**DOI:** 10.3390/ma14185157

**Published:** 2021-09-08

**Authors:** Eglė Kumpikaitė, Eglė Lapelytė, Stasė Petraitienė

**Affiliations:** 1 Department of Production Engineering, Faculty of Mechanical Engineering and Design, Kaunas University of Technology, Studentų Str. 56, LT-51424 Kaunas, Lithuania; eglelap@gmail.com; 2 Department of Applied Mathematics, Faculty of Mathematics and Natural Sciences, Kaunas University of Technology, Studentų Str. 50, LT-51424 Kaunas, Lithuania; stase.petraitiene@ktu.edu

**Keywords:** linen fabric, warp crimp, calculated crimp, experimental crimp

## Abstract

The aim of this study was to investigate the distribution of crimp in new jacquard fabric structures (in which one-layer and two-layer weaves are combined) in the fabric width and to create a method of crimp prediction. It was established that crimp was around 18.80% and changed within the limits of errors, i.e., a range of only ~4%, in the fabric width. It can therefore be said that the warp crimp was constant in the fabric width. Because the warp crimp of jacquard fabric changed insignificantly (within the limits of errors), it can be stated that the fabric-setting parameters and structural solutions were chosen and matched correctly, and such fabric can be woven on any jacquard weaving loom.

## 1. Introduction

There are several definitions of crimp. Percentage crimp is defined as the mean difference between the straightened thread length and the distance between the ends of the thread while in the cloth, expressed as a percentage [1]. The shortening of yarn length in a fabric is known as crimp [2]. When the warp and weft yarn interlace in a fabric, they follow a wavy path. This waviness of yarn is called crimp [3]. According to the definition of crimp, two values must be known: the length of cloth from which the yarns are removed and the straightened length of the thread. In order to straighten the thread, sufficient tension must be applied to remove all the kinks without stretching the yarn. In practice, it is seldom possible to remove all the crimp before the yarn itself begins to stretch. From these two values, the crimp percentage can be calculated with Equation (1):

(1) $$\mathrm{Yarn}\;\mathrm{Crimp}\;=100\cdot \frac{\mathrm{Straightened}\;\mathrm{Yarn}\;\mathrm{length}\;-\;\mathrm{Yarn}\;\mathrm{length}\;\mathrm{in}\;\mathrm{fabric}}{\mathrm{Fabric}\;\mathrm{Length}}$$

Warp and weft crimp percentage are two factors that have an influence on a fabric’s abrasion resistance, shrinkage, and fabric behaviour during strength testing [3].

Factors that affect crimp include the following:

1. Physical properties such as elasticity, rigidity, bending behaviour, etc. of fibres and yarns;
2. Count of warp and weft threads;
3. Setting of the threads;
4. Tension on the threads during weaving;
5. Yarn and fabric structure;
6. Physical and chemical treatment of the fabric after weaving [3].

Crimp frequency, amplitude, crimp stability, crimp elongation, and crimping point are some of the important properties that determine crimp. A fibre’s crimp characteristics have a strong influence on the processing performance of the fibres [3].

The construction of the warp and weft yarns are determined by various factors, such as the type and setting of weaving loom, the weaving conditions (moisture amount, temperature, yarn tension), the fabric weave, linear densities, the raw material of the threads, the flexibility of the warp and weft, and the pressure strength of the warp and weft threads in the positions of the floats. Thread crimp changes during weaving, fabric relaxation, and finishing [4]. Recently, it has been noticed that the thread diameter in the float positions and beat-up force can be attributed to these parameters [5].

Warp and weft crimp is important because it has an influence on weaving technology, warp and weft expenditure, fabric shrinkage during weaving and fabric properties (warp and weft breaking force, elongation at break, shape stability) [6].

During warping, it is important that all warp threads warped on the warp beam are equally taut. This is to ensure that the fabric’s properties during weaving do not change and remain the same throughout the entire fabric length. Without ensuring the same warp system tension across the whole width of the fabric, unequal fabric properties will be obtained. This not only affects the weaving process but can also negatively influence the weaving loom. Uneven tension in the thread system unequally affects their crimp and its distribution in the fabric [7].

Because warp and weft threads are oriented in two directions in the fabric, stretching fabric in the directions of warp and weft lengthens or widens the fabric in the direction of tension and shrinks it in the opposite direction. Elongation of the fabric is directly related to the decrease in crimp shrinkage in the same direction. Threads lengthen in the direction of load after removal of crimp, and the opposite thread system gains higher crimp. This will increase until one thread system does not interfere with the other [8].

Topalbekiroglu and Kaynak performed research [9] in which they investigated how fabric weaves influence a fabric’s stability. At first, they established that fabrics that have short floats in their weaves are more stable. If the weaves have longer floats, applying higher crimp can avoid instability in the fabric shape. This means that to have stable fabric, correct choice of certain weaves is needed, as well as ensuring optimal crimp [9].

Crimp also influences the fabric’s strength. Any modification of the fabric structure, which spreads the friction over a larger area improves abrasion resistance. Abrasion resistance increases when the friction load affects a larger number of threads and fibre removal or transfer from the fabric during friction decreases. Of course, floating threads, which are higher in comparison with the fabric surface, will suffer the most from abrasion load. In order to save the fabric’s tensile properties, fabric abrasion resistance should be increased; fabric appearance and/or mass loss are secondary factors. By increasing fabric crimp, abrasion resistance decreases. It is thought that this occurs because the crimp is lower when length of floats is higher. Fabric weaves and high crimp values, which would make some floats higher than others, thus concentrating abrasion loads, are not wanted. The locations of these floats would become the main weak links in the whole fabric [6].

The aim of Stig‘s and Hallström‘s research [10] was to find out how fabric weave and crimp influence rigidity and strength in 3D textile structures. It was established that rigidity and strength decrease non-linearly when crimp increases. The final strength of specimens with straight threads and of specimens with waved threads differed and noticeable stresses were significantly higher in threads that are waved by crimp. Specimens which contained crimped threads are weaker than those which are not crimped. Inserting perfectly straight threads into 3D fabric increases composite stiffness and strength, but some of the fibre volume also increases. However, the inserted threads are straight and stiffer than the warp threads, which are not waved by crimp. Because of this, they reach maximal stresses under low loads [10]. This phenomenon influences the decrease in 3D fabric strength in the longitudinal direction and the increase in stiffness in the longitudinal direction.

The aim of this paper was to investigate the distribution of crimp in jacquard fabrics across the fabric width and to create a method of predicting crimp in jacquard fabrics.

## 2. Experimental Procedure

**Figure 1:**
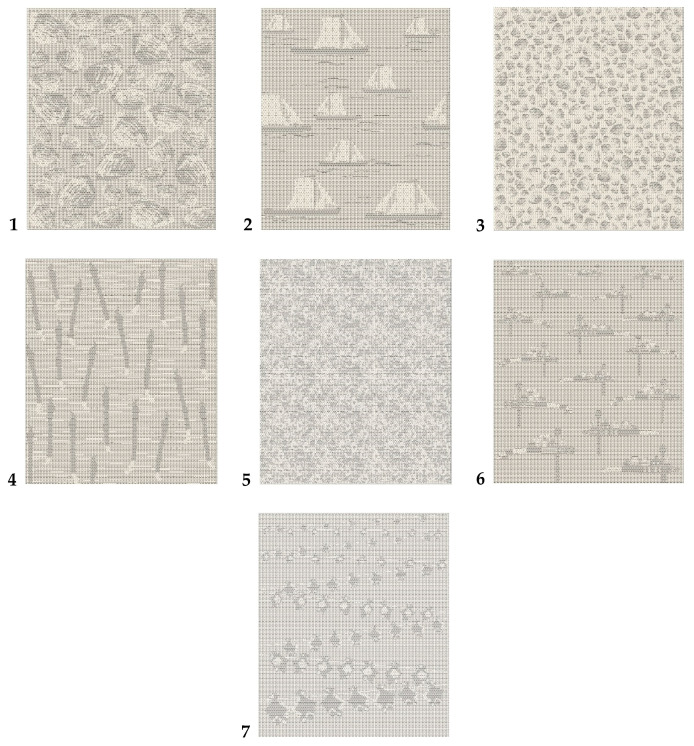
Designs of the jacquard fabrics used for the experiments: **1**—big shells; **2**—ships; **3**—small shels; **4**—birds; **5**—crystals of sand; **6**—weathervanes; **7**—dried fish.

New jacquard fabric structures, in which one-layer and two-layer weaves are combined, were woven by the joint-stock company Lincasa on a P1 rapier weaving loom (Lindauer Dornier GmbH, Lindau, Germany). The warp and weft yarns were linen; their linear density was 50 tex. They were single-ply yarns with 523 m^−1^ twist in the Z direction, the breaking force was 5.286 N and the elongation at break was 1.7%. The warp setting was 21 ends/cm and the weft setting was 16 picks/cm. In the weft direction, every second thread was bleached and every other second thread was of a natural colour. The warp was bleached. The number of jacquard machine hooks was 3360. The fabric width in the reed was 160 cm. The initial warp tension was 7.43 mN/tex, the healds’ cross-advance was at 10 degrees of the main loom shaft. Fabrics were woven in seven different designs (Figure 1).

**Figure 2:**
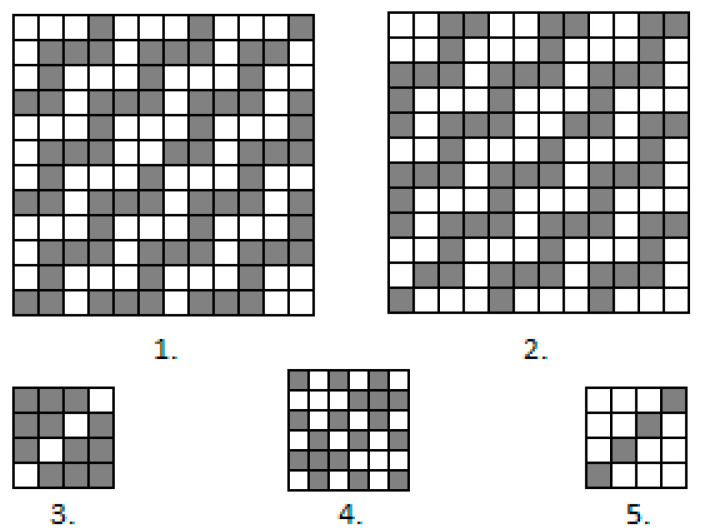
Weaves of used Jacquard fabrics: **1**—weave of dark grey colour in fabric design; **2**—weave of greyish colour in fabric design; **3**—weave of light grey colour in fabric design; **4**—weave of background in fabric design; **5**—weave of grey colour in fabric design.

The dimensions of all designs were 1.5 m × 2.0 m. All the designs were woven using five different weaves, which are presented in Figure 2. Weaves 1 and 2 used dark grey and greyish in all designs. These weaves were similar but they began from different threads. For this reason, the bleached weft dominated in Weave 1 and the natural-colour weft dominated in Weave 2. The background of all designs was woven in Weave 4. Weaves 3 and 5 were used for pattern highlighting and correspond to two other shades of grey in the designs.

**Figure 3:**
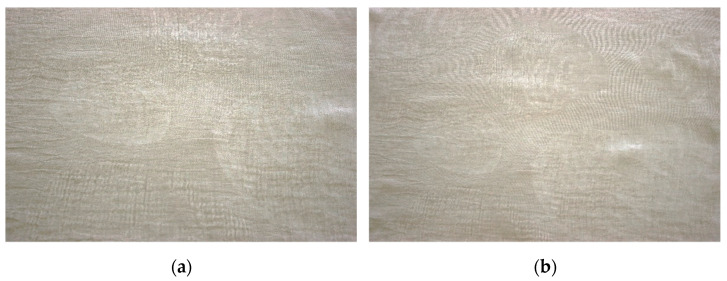
Fragment of jacquard fabric Design 1: (**a**) from the right side; (**b**) from the wrong side.

Examples of fragments of jacquard fabric Design 1 from the right (Figure 3a) and wrong (Figure 3b) sides are presented in Figure 3. To show a general view of the design would make no sense, because the dimensions of the design are very big and the differences between different weaves and colours do not emerge in pictures of the entire design. The same situation occurs with the other six designs.

A known problem of all jacquard fabrics’ weavability is that the weaving design has to be such that uniform warp yarn crimp over the entire width of the fabric can be achieved. This problem exists in every jacquard fabric but this question is particularly relevant when combining one-layer and two-layer weaves in one fabric. In order to distribute one-layer and two-layer weaves evenly in the fabric’s width, warp crimp was established in different places of the fabric width. A random part of the fabric length was chosen and a segment of a certain length was measured. We tried to ensure that this place was not the beginning of the repeat and that the fabric repeat did not finish in the whole segment. The fabric was divided into six approximately equal parts, distributed equally across the fabric width. When the fabric had been divided into separate specimens, 10 warp threads were unpicked in the right side of the specimen. Their length was measured. Equation (2) was used for calculating the crimp:

(2) $$a=\frac{l_{s}-l_{aud}}{l_{s}}\cdot 100\%$$

where: *l_s_* is the length of the warp thread and *l_aud_* is the length of fabric specimen.

## 3. Results and Discussion

When weaving jacquard fabrics, it is very important that the warp crimp in different places in the fabric’s width should be the same or differ insignificantly. In such a case, the jacquard fabric’s weavability will be good. In other cases, some warp yarns or their groups will be stretched and other ones will be loose. This fact is especially important in fabrics in which one-layer and two-layer weaves are combined. However, the investigation of fabric crimp across the width is very relevant to all jacquard fabrics.

**Figure 4:**
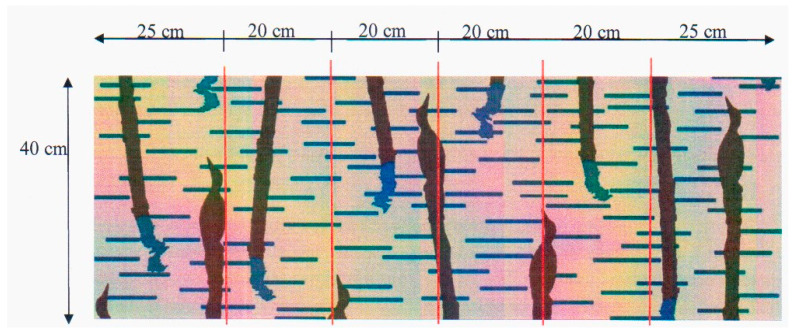
Division of the fabric section.

In order to investigate crimp distribution in the fabric’s width, warp crimp in various parts of the fabric was established. A random location in the fabric length was chosen and a segment 40 cm in length was measured. The fabric was divided into six parts, which were distributed across the fabric width as follows: 25 cm, 20 cm, 20 cm, 20 cm, 20 cm, 20 cm, and 25 cm. A schematic view of specimen partitioning is shown in Figure 4.

After dividing the fabric into separate specimens, 10 warp threads were unpicked from the right side of each specimen. The length of these 10 threads was measured. The crimp of the warp threads was calculated according to Equation (2).

**Figure 5:**
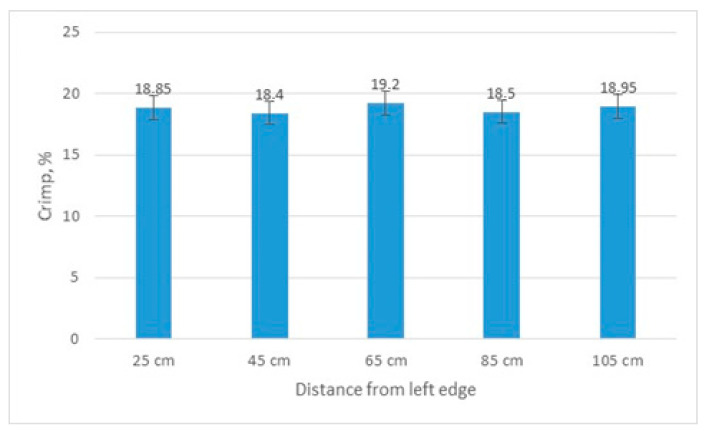
Results of fabric crimp calculation across the fabric width.

The distribution of crimp across the fabric width is demonstrated in Figure 5. It can be seen that the differences in crimp in different places across the fabric width are insignificant: they differ by up to 4% (18.40–19.20%), i.e., they vary within the error margin, because the variation coefficient is up to 5.4%. Thus, it can be stated that the warp crimp is almost equal in different places of the fabric. The conclusion can be drawn that the fabric design has been made correctly, as the locations of different weaves were distributed evenly throughout the entire fabric repeat.

In order to explain how crimp changes in parts of fabric woven in different weaves, an original method is suggested. By applying this method, the crimp was calculated in certain segments. Specimens 10 cm in length were manufactured in which sections woven with different weaves were found. In the case of new Jacquard fabric structures, the parts woven in one-layer and two-layer weaves were analysed separately, because the float distribution inside these groups was similar. Ten different warp threads were pulled out from four different specimens and the crimp was calculated according to Equation (2) separately for one-layer and two-layer fabric sections.

The crimp of one-layer weaves should be the same because the floats’ lengths and their distribution are the same in one-layer weaves; only the character of the floats differs. The same situation also occurs with two-layer weaves because the floats are only shifted through a single thread and the floats’ distribution in the weave are the same. The results of calculating the crimp for one-layer and two-layer weaves are presented in Table 1. In the case of another jacquard fabric structure, the number of columns in the table will correspond to the number of different weaves in the fabric. The dispersion of the results is very low, i.e., the coefficient of variation for one-layer weaves is just 0.17% and for two-layer weaves, it is 0.19%. Thus, the accuracy of crimp calculation is very high.

In order to calculate the crimp, we determined the percentage of warp threads interwoven in one-layer and two-layer weaves along their length in the specimen. In the case of another jacquard fabric structure, the percentage of warp threads interwoven in each different weave will be calculated. Adobe Photoshop CS6 software (version 13.1x, Adobe Systems Incorporated, San Chose, CA, USA), which allowed us to visualise the created fabric at the pixel level, was used for calculating this parameter. It was established during this investigation that one pixel equals one warp thread. Next, a newly created jacquard fabric design was divided across the entire fabric width into the same six parts as the real fabric (25 cm, 20 cm, 20 cm, 20 cm, 20 cm, 20 cm, and 25 cm), the length of which is 40 cm. Ten pixel columns from the specimen’s right side were calculated. The proportion of one-layer and two-layer weaves in separate warp threads is presented in Table 2.

When proportion of one-layer and two-layer weaves in the chosen warp threads is known, the crimp of chosen warp threads can be calculated according to Equation (3):

(3) $$a_{sk}=\frac{d_{1-sl}a_{1-sl}+d_{2-sl}a_{2-sl}}{100}$$

where $d_{1-sl}$ is the proportion of one-layer weaves in a certain warp thread (%), $a_{1-sl}$ is the crimp of one-layer weaves (from Table 2), $d_{2-sl}$ is the proportion of two-layer weaves in a certain warp thread (%) and $a_{2-sl}$ is the crimp of two-layer weaves (from Table 2).

Moreover, $d_{1-sl}$ and $d_{2-sl}$ were calculated according to Equations (4) and (5):

(4) $$d_{1-sl}=\frac{n_{1-sl}}{n_{sum}}\cdot 100\%$$

(5) $$d_{2-sl}=\frac{n_{2-sl}}{n_{sum}}\cdot 100\%$$

where $n_{1-sl}$ is the number of pixels in the column corresponding to number of threads woven in one-layer weaves along the warp thread in the jacquard pattern draft, $n_{2-sl}$ is the number of pixels in the column corresponding to number of threads woven in two-layer weaves along the warp thread in the draft jacquard pattern, and $n_{sum}$ is the number of pixels in one column of the analysed part of the draft jacquard pattern.

General warp crimp consists of a few one-layer and a few two-layer weaves, and their proportions in a jacquard fabric are evaluated in Equation (3), when crimps of each separate weave are known. Thus, the warp crimp of each separate warp thread in jacquard fabric can be predicted according to Equation (3) just by looking at the draft fabric pattern.

If the jacquard fabric is woven using another weave and structure, Equations (3)–(5) can be rewritten as:

(6) $$a_{skb}=\frac{d_{1}a_{1}+d_{2}a_{2}+\cdots +d_{n}a_{n}}{100}$$

(7) $$d_{1}=\frac{n_{1}}{n_{sum}}\cdot 100\%$$

(8) $$d_{2}=\frac{n_{2}}{n_{sum}}\cdot 100\%$$

(9) $$d_{n}=\frac{n_{n}}{n_{sum}}\cdot 100\%$$

where $d_{1}$ is the proportion of the first weave in a certain warp thread (%), $a_{1}$ is the crimp of the first weave, $d_{2}$ is the proportion of the second weave in a certain warp thread (%), $a_{2}$ is the crimp of the second weave, $d_{n}$ is the proportion of the *n*th weave in a certain thread (%), $a_{n}$ is the crimp of the *n*th weave, $n_{1}$ is the number of pixels in the column corresponding to number of threads woven in the first weave along the warp thread in the draft jacquard pattern, $n_{2}$ is the number of pixels in the column corresponding to the number of threads woven in the second weave along the warp thread in the draft jacquard pattern, $n_{n}$ is the number of pixels in the column corresponding to number of threads woven in the *n*th weave along the warp thread in the draft jacquard pattern, and $n_{sum}$ is the number of pixels in one column of the analysed part of the draft Jacquard pattern.

The results of calculating the crimp are demonstrated in Table 3. The coefficients of variation of the experiments, which vary from 0.14% to 0.16%, show the high accuracy and low dispersion of the calculated crimp.

**Figure 6:**
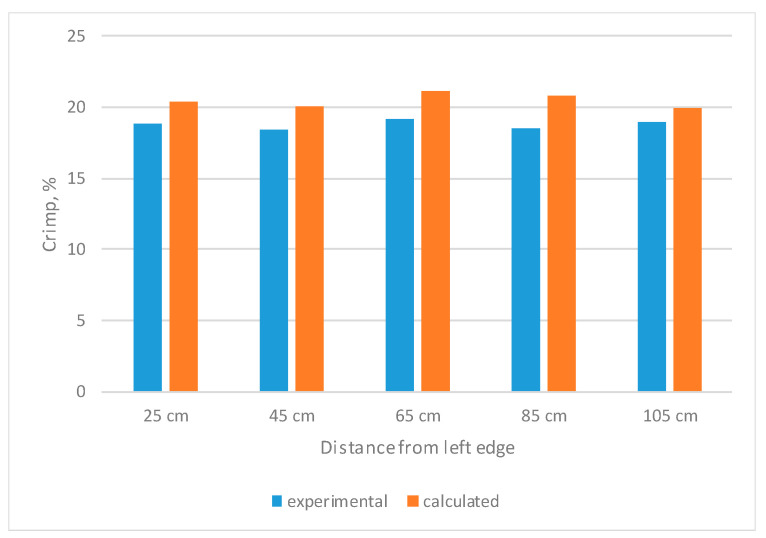
Comparison of experimental and calculated crimp.

In a comparison between the experimental and calculated crimp results, comparable crimp results are shown in Figure 6. As can be seen from the diagram, the calculated crimp is higher than the experimental crimp in all cases by 5% for 105 cm distance from the fabric edge to 10% for 65 cm distance from the fabric edge. Such results were obtained because the crimp of separate weaves was established from shorter segments of the warp threads, which were woven in such weaves. Because of that, the accuracy of crimp could have been established differently.

The results showed that warp crimp changed within the limits of errors in the jacquard fabric’s width. Thus, it can be stated that the fabric’s structural parameters and structural solutions had been chosen correctly.

The same investigation of crimp was performed with seven different jacquard fabrics patterns woven with the same weaves but with different distributions in the fabric. The results are presented in Table 4. The relative error of the experimental crimp varied from 7.86% to 12.35% and the relative error of the calculated crimp varied from 6.06% to 12.41%. Thus, it can be stated that the accuracy of the results is sufficient.

As can be seen from Table 4, the distributions of warp crimp in fabrics with different distributions of weaves are similar. Thus, it can be stated that the suggested method of predicting warp crimp in the weave structure described is correct. It can be used for jacquard fabrics of any structure.

## 4. Conclusions

After evaluation of jacquard fabrics’ crimp in the warp direction, it was established that the crimp was 18.80% and changed within the limits of errors, i.e., a range of only 4% percent, in the fabric width. Thus, it can be said that the warp crimp was constant across the fabric width.

Because the warp crimp of Jacquard fabric changed insignificantly (within the limits of errors), it can be stated that the fabric’s setting parameters and structural solutions were chosen and matched correctly and such a fabric could be woven on any Jacquard weaving loom.

After a comparison of the experimental and calculated warp crimps, it was established that calculated crimp in all cases was higher than the experimental crimpy by 5–10% across the fabric width. The reason for this may be the lower accuracy of establishing the calculated crimp, because the crimp of different weaves was established from shorter segments of the warp threads.

## Figures and Tables

**Table 1:** Results of calculating the crimp for one-layer and two-layer weaves.

Number	Crimp of One-Layer Weaves%	Crimp of Two-Layer Weaves%
1	15.25	21.88
2	23.08	18.70
3	23.08	28.57
4	16.67	18.70
5	20.00	25.92
6	23.08	26.47
7	16.67	20.63
8	24.24	21.88
9	19.35	20.00
10	18.03	20.00
11	19.35	19.35
12	25.37	25.37
13	19.35	19.35
14	18.03	18.03
15	16.67	16.67
16	18.70	18.70
17	20.63	20.63
18	18.03	18.03
19	18.70	18.70
20	11.50	11.50
Average	19.29	20.45
Standard deviation	3.32	3.85
Coefficient of variation	0.17	0.19
Histogram	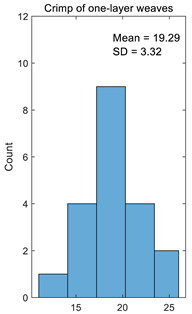	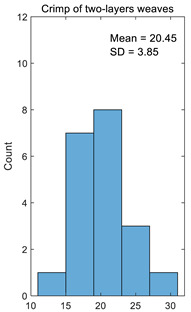

**Table 2:** Proportion of one-layer and two-layer weaves.

Number	25 cm from Edge		45 cm from Edge		65 cm from Edge		85 cm from Edge		105 cm from Edge	
	1-l. We. Part%	2-l. We. Part%	1-l. We. Part%	2-l. We. Part%	1-l. We. Part%	2-l. We. Part%	1-l. We. Part%	2-l. We. Part%	1-l. We. Part%	2-l. We. Part%
1	66.2	33.8	97.6	2.4	45.2	54.8	62.2	37.8	100	0
2	73.8	26.2	97.6	2.4	44.2	55.8	63.1	36.9	100	0
3	79.0	21.0	97.9	2.1	47.2	52.8	63.8	36.2	100	0
4	82.1	17.8	97.9	2.1	46.8	53.2	64.0	36.0	100	0
5	88.1	11.9	98.1	1.9	45.0	55.0	65.0	35.0	100	0
6	92.1	7.8	98.1	1.9	47.4	52.6	65.4	34.6	100	0
7	93.4	6.6	98.2	1.8	48.6	51.4	65.8	34.2	100	0
8	94.9	5.1	98.3	1.7	49.4	50.6	66.3	33.7	100	0
9	95.2	4.8	98.5	1.5	46.5	53.5	67.4	32.6	100	0
10	98.2	1.4	98.7	1.3	46.0	54.0	67.4	32.6	100	0

**Table 3:** Results of calculating the crimp.

Number	25 cm from Edge%	45 cm from Edge%	65 cm from Edge%	85 cm from Edge%	105 cm from Edge%
1	17.49	15.41	18.89	17.75	15.25
2	21.93	22.98	20.64	21.46	23.08
3	24.23	23.20	25.98	25.07	23.08
4	17.03	16.71	17.75	17.40	16.67
5	20.70	20.11	23.26	22.07	20.00
6	23.35	23.15	24.86	24.25	23.08
7	16.93	16.74	18.71	18.02	16.67
8	24.12	24.20	23.05	23.44	24.24
9	19.38	19.36	19.70	19.56	19.35
10	18.07	18.06	19.09	18.67	18.03
Average	20.32	19.99	21.19	20.77	19.95
Standard deviation	2.94	3.22	2.87	2.86	3.26
Coefficient of variation	0.14	0.16	0.14	0.14	0.16
Histogram	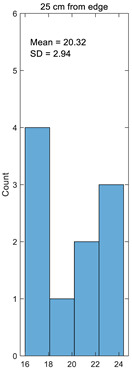	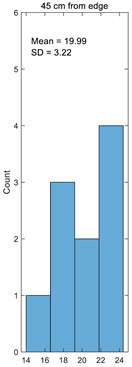	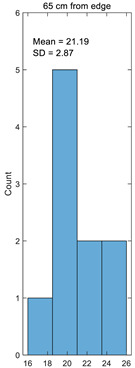	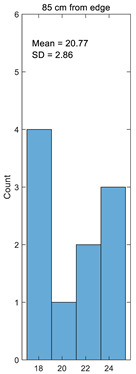	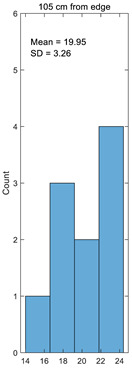

**Table 4:** Results of the experimental and calculated crimp of different fabrics.

Design Number	Experimental Crimp%	Calculated Crimp%
1	19.04 ± 2.23	20.05 ± 1.32
2	18.58 ± 2.04	19.96 ± 1.21
3	19.12 ± 1.40	20.14 ± 2.50
4	19.08 ± 2.35	21.10 ± 1.87
5	18.44 ± 1.45	20.57 ± 1.44
6	18.63 ± 1.89	20.92 ± 2.34
7	18.78 ± 2.32	20.24 ± 2.41

## Data Availability

Data are contained within this article.
